# Sintering, Mechanical and Optical Properties of TiB_2_ Composites with and without High-Energy Milling

**DOI:** 10.3390/nano13192683

**Published:** 2023-09-30

**Authors:** Simone Taraborelli, Simone Failla, Elisa Sani, Diletta Sciti

**Affiliations:** 1 Institute of Science, Technology and Sustainability for Ceramic, National Research Council of Italy, Via Granarolo 64, I-48018 Faenza, Italy; simone.failla@issmc.cnr.it; 2 Department of Chemistry, Life Sciences and Environmental Sustainability, University of Parma, Parco Area delle Scienze 11/a, I-43124 Parma, Italy; 3 National Institute of Optics, National Research Council of Italy, Largo E. Fermi, 6, I-50125 Firenze, Italy; elisa.sani@ino.cnr.it

**Keywords:** TiB_2_, high-energy milling, hardness, armor materials, optical properties, solar absorbers

## Abstract

TiB_2_ is a promising material for several fields including impact-resistant armor, wear-resistant coatings, cutting tools and crucibles given its physical, mechanical and chemical properties, especially due to the combination of high hardness and exceptional wear resistance. It is however very difficult to sinter below 2000 °C, even under mechanical pressure; moreover, the low fracture toughness limits the applicability of the ceramic material. By using sintering additives, it is possible to improve the sintering process and increase the mechanical properties since the additives react with oxidized layers and form secondary phases. In this study, different preparation methods and various combinations of additives (B_4_C, Si_3_N_4_ and MoSi_2_) via hot pressing sintering have been explored. Through the synergy between optimized process and tailored composition, an almost fully dense material was obtained at 1700 °C with hardness of 24.4 ± 0.2 GPa and fracture toughness of 5.4 ± 0.2 MPa m^1/2^. However, the highest hardness (24.5 ± 0.2 GPa) and density values were obtained for only the high-energy-milled sample with WC-Co media, featuring a core–shell grain structure. Finally, optical properties for selected samples were measured, identifying the high-energy-milled TiB_2_ as the sample with the highest spectral selectivity α/ε and solar absorptance.

## 1. Introduction

Among non-oxide ceramics, TiB_2_ [1,2,3,4,5,6,7,8,9,10] is particularly studied for several reasons. Firstly, it is a hard and stiff ceramic. Moreover, it possesses a very high melting point (3225 °C) [11] and exhibits a level of electrical conductivity similar to that of metals [12]. This feature can be effectively utilized in electro-discharge machining, which is a more cost-effective technique compared to conventional diamond tool machining for shaping ceramic components.

Despite the numerous advantages of using TiB_2_, achieving a fully dense material is highly challenging. This is primarily due to the low self-diffusion coefficient and relatively high vapor pressure of its constituents, [13] resulting from the presence of both ionic and covalent bonds [14]. Furthermore, the presence of an oxide layer (TiO_2_ and B_2_O_3_) on the surface of TiB_2_ powder promotes excessive grain growth through vapor or surface transport mechanisms during the sintering process, leading to the formation of porosity that becomes trapped within the grains [15,16].

For all the previously addressed issues, among the possible densification routes, pressureless sintering (PLS) of TiB_2_ seems to be an unattractive choice, since it requires relatively high temperatures (2000–2200 °C) and many hours of dwell time to achieve a good densification. This can lead to exaggerated grain growth and consequently to lower mechanical properties due to the anisotropy of the hexagonal grain structure that results in deleterious internal stresses [6,15]. Grain growth can also cause microcracking when average grain size exceeds a critical value, which experimental observations suggest to be ~15 µm [14,17,18,19,20].

Densification by hot pressing (HP) can lower the sintering temperature (1800–2000 °C), increase the sintering rate and achieve a uniform microstructure of sintered materials [13,21,22,23,24]. Spark plasma sintering (SPS) is an alternative strategy where the combination of applied external pressure and current with a very high intensity leads to rapid heating, lower working temperature and shorter dwell time. The advantages of the HP and SPS techniques are a higher density and finer final microstructure. However, both processes are limited by a simple sample geometry [10,25,26,27,28,29,30].

One more way to improve the sintering of TiB_2_ is through addition of sintering aids, that can be either metallic or non-metallic. Metallic additives like Ti [31], Co [32], Cr [33], Ni [34], Fe [35], Al [36] and Ta [37] may lead to high relative density through liquid phase sintering (LPS); indeed, these transition metals can react with TiB_2_ and B_2_O_3_ to form various metal borides with a low melting point and a good wetting behavior. Although the liquid phase increases mass transport and decreases the sintering temperature (1500–1700 °C), accelerated grain growth is observed [38,39,40]. Moreover, metallic additives have been found to reduce the corrosion resistance, wear properties and high temperature properties of TiB_2_ [14,26,40,41].

Therefore, non-metallic additives such as carbides (B_4_C [42], SiC [43], TiC [44], TaC [45], WC [46]), nitrides (Si_3_N_4_ [9], AlN [10,47], TiN [29,43]), disilicides (TiSi_2_ [48], MoSi_2_ [7,49,50,51]) and borides (ZrB_2_ [52]) are preferred for applications at elevated temperatures.

These additives react with the oxide surface layer of TiB_2_ particles and form secondary phases, improving sinterability without promoting grain growth and possibly leading to lower sintering temperatures (1700–2000 °C) and better mechanical properties [26]. On the other hand, the production of secondary phases may result in a significant reduction in purity of the TiB_2_, thus, leading to a decrease in electric conductivity and thermal conductivity [25,47].

The purpose of this work is to optimize the densification of TiB_2_ by hot pressing and introducing small amounts of B_4_C, Si_3_N_4_ or MoSi_2_ as a sintering aid. In addition, the effect of high-energy milling with WC milling beads is also considered, in combination or not with these sintering aids. Densification mechanisms and microstructural characterization are carefully considered. In addition, hardness and toughness measurements were carried out. The optical properties of TiB_2_ ceramics were also investigated in order to explore their possible application as solar absorbers. In fact, a single previous study on the optical properties of TiB_2_ bulks can be found [53], which reported a higher solar absorptance with respect to other monophasic borides (ZrB_2_ and TaB_2_) and a comparable spectral selectivity with respect to MoSi_2_-added dense ZrB_2_ and TaB_2_, definitely suggesting that TiB_2_ is worthy of further studies for its use in solar thermal technologies. The cited work dealt with a monophasic and fully dense TiB_2_ pellet. However, it is known that the material composition with the possible presence of additives and byproducts, as well as the porosity, have a critical influence on the overall optical properties of UHTC ceramics [54,55,56,57].

## 2. Materials and Methods

Commercial powders of TiB_2_ (H.C. Starck, Grade F, D_90_ 4.0–7.0 μm, D_50_ 2.5–3.5 μm, <4.5 μm, impurities (wt. %): 0.4 C, 2.5 O, 0.5 N, 0.1 Fe), were used to produce powder mixture. The following commercial powders were used as additives:

- B_4_C (H.C. Starck Grade HS-A, D_90_ 2.0–4.0 μm, D_50_ 0.6–1.2 μm, B:C ratio 3.7, impurities (wt. %): 0.7 N, 1.7 O, 0.05 Fe, 0.15 Si, 0.05 Al);
- Si_3_N_4_ (α-Si_3_N_4_, H.C. Starck Grade M 11, α > 90%, D_50_ 0.6 μm, D_90_ 1.3 μm, D_10_ 0.3 μm, impurities (wt. %): 0.5 O, 0.5 C, 0.08 Al, 0.01 Ca, 0.008 Fe);
- MoSi_2_ (Sigma Aldrich, purity > 99% average particle size < 2 μm, metallic impurities (<2000 PPM): 400 ppm Al, 12 ppm Ba, 16ppm Cr, 400 Fe, 12 ppm K, 39 ppm W).

Preliminarily, the as-received TiB_2_ powder was milled via high-energy milling (HEM) in a WC jar using 0.5 mm WC-6Co spheres in air for 10, 20 and 30 min to analyze the change in the particle size distribution. Each ten minutes, small powder batches were analyzed using the sedimentation method (SEDIGRAPH III plus, Micromeritics) to determine the particle size distribution of the baseline TiB_2_ (with no sintering additives) with scanning electron microscopy (FE-SEM, Carl Zeiss Sigma NTS GmbH, Oberkochen, Germany) equipped with an energy dispersive microanalysis (EDS, Model INCA energy 300; Oxford Instruments, Abingdon, UK) system to qualitatively track the main contaminants and XRD (Bruker D8 Advance, Karlsruhe, Germany).

Then, powder mixtures were prepared according to the following compositions:

- TiB_2_ + 5 vol.% B_4_C, labeled as TC;
- TiB_2_ + 5 vol.% Si_3_N_4_, labeled as TS;
- TiB_2_ + 5 vol.% MoSi_2_, labeled as TM.

For each composition, one batch was soft homogenized by ball milling in ethyl alcohol (EtOH), and the second one was additionally milled via high-energy milling for 30 min (labeled as TC-h, TS-h, TM-h). Two additional batches were the as-received TiB_2_ and the high-energy-milled TiB_2_ (labeled as T and T-h).

The eight powder mixtures were then sintered in a graphite mould using an induction-heated hot-pressing machine at 1700–1950 °C, with applied pressure of 30–35 MPa and holding time of 10 min in a vacuum (0.1–0.2 mbar).

X-ray diffraction analysis was carried out on dense samples to determine the crystalline phases. After sintering, the bulk densities were measured using Archimedes’ method. The theoretical densities of the samples were calculated using the rule of mixtures taking into account the incorporated WC and Co phases.

Fracture and polished sections were analyzed with SEM-EDS. Image analysis (Image-Pro Plus^®^ version 7, Media Cybernetics, Silver Springs, MD, USA) carried out on the polished sections was used to determine the mean grain size of the sintered samples.

As for the mechanical properties, the hardness was tested with Vickers indentation, with a load of 1 Kg (Innovatest Falcon 505, Rupac, The Netherlands). Young’s modulus was determined through the resonance frequency method.

Spectra were collected on unpolished sample cross-sections cut with diamond blades, ground, and carefully cleaned. Optical reflectance spectra at room temperature in the 0.25–2.5 µm wavelength region were acquired using a double-beam spectrophotometer (Lambda900, Perkin Elmer, Waltham, Massachusetts) equipped with a 150 mm diameter integration sphere for the measurement of the hemispherical reflectance. The spectra in the wavelength region 2.5–15.5 µm were acquired using a Fourier Transform spectrophotometer (FT-IR Excalibur, Bio-Rad, Hercules, California) equipped with a gold-coated integrating sphere and a liquid-nitrogen-cooled detector. In all cases, the reflectance spectra were acquired for a quasi-normal incidence angle.

## 3. Results and Discussion

### 3.1. Effect of Milling with WC-Co Beads

**Figure 1:**
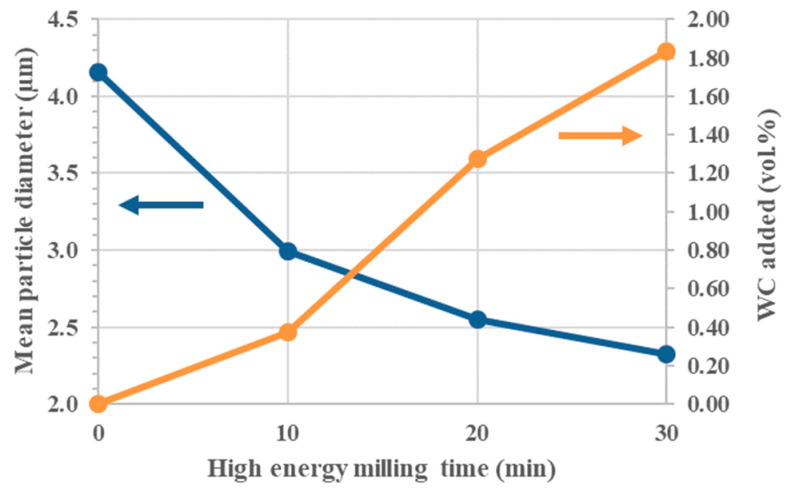
Mean particle diameter and WC-Co amount added after 10, 20 and 30 min of HEM.

**Figure 2:**
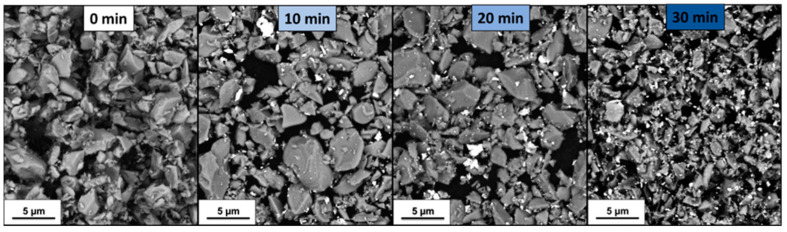
Backscatter electron micrographs of TiB_2_ powders before and after 10, 20 and 30 min of HEM.

The amount of incorporated WC was determined by weighing the powders before and after the milling procedure. The plot of Figure 1 shows a significant decrease in mean grain size and an increase in WC-Co contamination (~1.8 vol.%) after 30 min of high-energy milling. The SEM images in Figure 2 confirm the progressive decrease in the size of TiB_2_ particles and the introduction of submicrometric particles of WC-Co depicted as white particles. The reduction rate of the mean particle diameter decreased progressively during HEM without reaching a plateau, while the mass from the grinding media seems to have increased linearly within the first 30 min.

**Figure 3:**
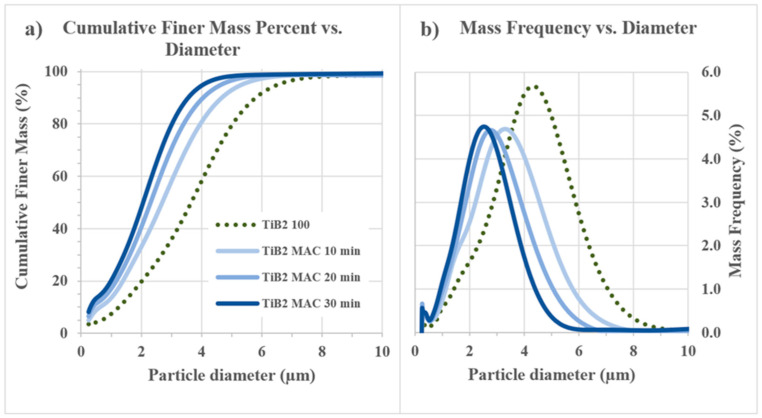
Cumulative finer mass percent vs. diameter (**a**) and mass frequency vs. diameter (**b**) for TiB_2_ particles before and after 10, 20 and 30 min HEM.

Furthermore, HEM resulted in a progressive increase in the number of particles with a diameter below 0.5 µm. The sharp peak observed in the distribution of Figure 3 was attributed to the lower limit of resolution, which prevented the distinction of particles with a diameter of 0.25 microns or less. This final peak was caused by the submicrometric particles of WC-Co introduced during HEM, and it increased as the milling process progressed.

The coarse fraction of the mixture was reduced, as evidenced by the change in the D90 value of the starting TiB_2_ from the nominal 5.8 µm (the coarsest) to 3.6 µm in the 30 min planetary milled mixture. It was found that TiB_2_ has a significant amount of oxygen impurities. According to previous analyses on B_4_C-TiB_2_ composites, SEM/TEM images of the powder mixtures after milling revealed that TiB_2_ particles were surrounded with a continuous amorphous layer of about 5.5 ± 1 nm, containing Ti, W and O elements [58]. SEM analyses confirmed that high-energy milling resulted in smaller particle sizes and contamination with WC/Co-Cr due to wear of WC-Co balls. This contamination could be attributed to a mechanical alloying phenomenon [59].

Additionally, alloy nanoparticles with different chemical compositions, such as W-rich (W-Ti-Cr-Co) and Co-rich (Co-Cr-Ti-W), were observed to be attached to TiB_2_ due to the mechanical alloying process during high-energy milling. These nanoparticles were formed as a result of contamination from other metallic species, such as W, Co and Cr [58].

### 3.2. Sintering Behavior and Microstructural Features

The details of the sintering cycles and quantitative data on microstructural features are summarized in Table 1. The hot-pressing schedule was determined during the heating process based on the recorded shrinkage measured by the displacement of the rams. Due to the reactions between the matrix, sintering aids and oxide phase, it was not possible to calculate a theoretical density for the different compositions, except for the T sample, which consisted solely of as-received TiB_2_. The relative density of samples was calculated as (1-P) where P represents the percent porosity determined through image analysis on secondary electron micrographs of the polished sections.

**Figure 4:**
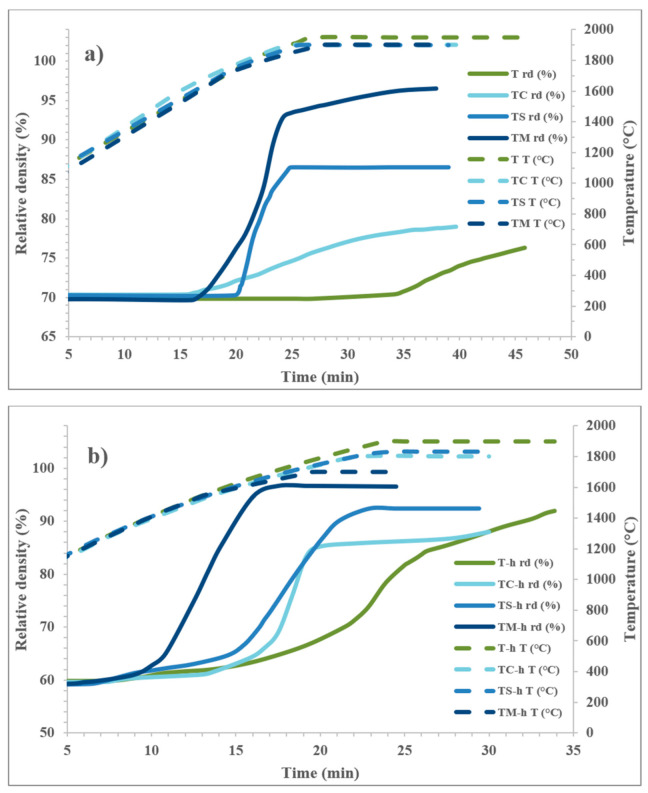
Comparison of densification curves for soft homogenized (**a**) and high-energy-milled (**b**) HP sintered samples.

The densification behavior was clearly affected by both the addition of sintering agents and the high-energy milling of the powders, as shown in Figure 4a,b which present the relative density plotted against time. From this type of graph, it can be observed at which temperature the shrinkage starts and the maximum temperature needed for complete densification.

It can be observed that for the as-received TiB_2_ powder, densification was very slow and only began after 10 min at 1950 °C. In contrast, for the powder mixtures containing the sintering additives, densification started at lower temperatures: 1630 °C for TC (addition of B_4_C), 1770 °C for TS (addition of Si_3_N_4_) and 1710 °C for TM-h (addition of MoSi_2_); see curve in Fig 4a. The addition of boron carbide increased densification rate, but the final density remained below 80%. Si_3_N_4_ and MoSi_2_ notably improved sintering, as indicated by a steep increase in the densification curve and the presence of SiO_2_ pockets after sintering for both samples. The steep change in the slope of the curve could be an indication of liquid phase formation and the increase in the densification rate due to particle rearrangement.

In the case of high-energy-milled powders, densification initiated at 1570 °C for T-h (reference, no sintering aids), 1530 °C for TC-h (addition of B_4_C), 1560 °C for TS-h (addition of Si_3_N_4_) and 1250 °C for TM-h (addition of MoSi_2_). For only the high-energy-milled TiB_2_ ceramic, the increase in the shrinkage rate was more gradual. In contrast, a steep increase similar to what was observed for the TS and TM samples was also noticed for the three systems with B_4_C, Si_3_N_4_ or MoSi_2_.

**Figure 5:**
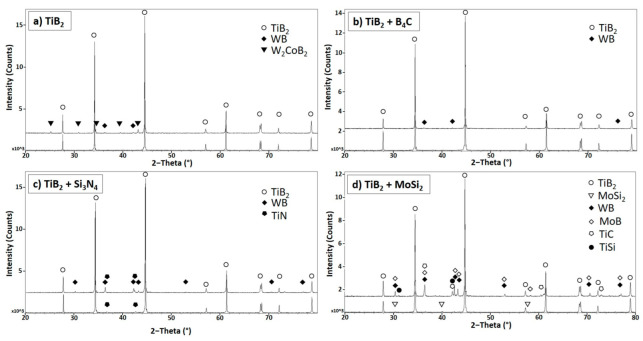
X-ray diffraction patterns for the soft homogenized (line below) and high-energy-milled (line above) sample of TiB_2_ without additives (**a**), with 5 vol.% of B_4_C (**b**), 5 vol.% of Si_3_N_4_ (**c**) and 5 vol.% of MoSi_2_ (**d**).

X-ray diffraction patterns (Figure 5) recorded TiB_2_ (TiB_2_: JCPDF card 35-0741) in all samples and WB for the high-energy-milled ones, along with minor secondary phases depending on the sintering aid and the preparation method employed. After sintering, no B_4_C or Si_3_N_4_ peaks were detected, while MoSi_2_ was still present in the softly homogenized sample. Moreover, TiN was observed in the Si_3_N_4_ samples (TS and TS-h) originating from the reaction between Si_3_N_4_ and TiO_2_.

W_2_CoB_2_ was only found in the high-energy-milled sample of TiB_2_ without additives (T-h), TM-h (MoSi_2_) exhibited phases of MoB, TiC and TiSi.

An overview of the microstructural features of both the ball-milled and high-energy-milled samples is provided in Table 1. It can be observed that all samples treated with high-energy milling displayed a higher degree of densification, and the highest densification grades were obtained for TiB_2_ samples densified with MoSi_2_ as a sintering aid (TM and TM-h), which reached final relative densities equal to 95.1% for the soft homogenized sample and 94.7% for the HEM sample.

A detailed description of the microstructure is given below.

T and T-h. The EDS spectrum of the three different phases confirmed the absence of the other metals in the TiB_2_ cores and the presence of W and Co in TiB_2_ solid solution rims. Moreover, the presence of Cr and C can be observed in the mixed boride from WC-Co grinding media: chromium was indeed present in lower quantities than Co, and carbon is attributable to residual unreacted carbide.

**Figure 6:**
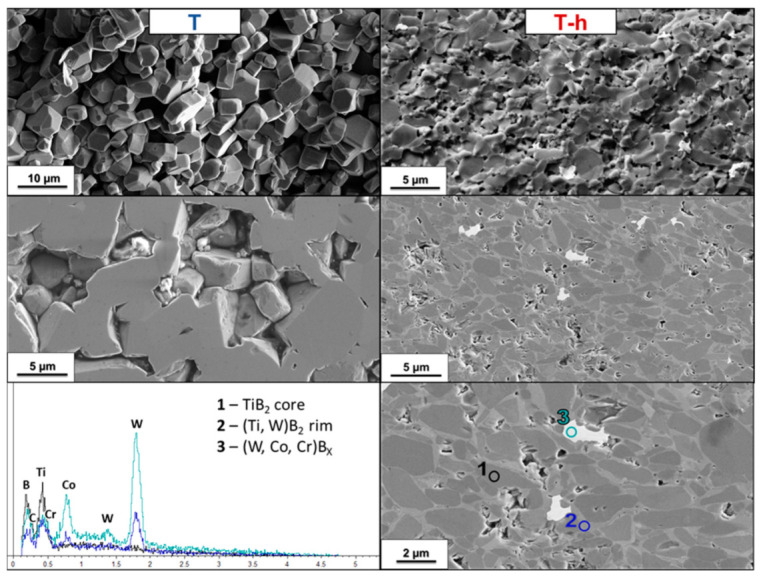
SEM micrograph of T (**left column**) and T-h (**right column**) samples’ fractured and polished sections and EDS spectrum of T-h sample’s polished section (**third row**).

**Figure 7:**
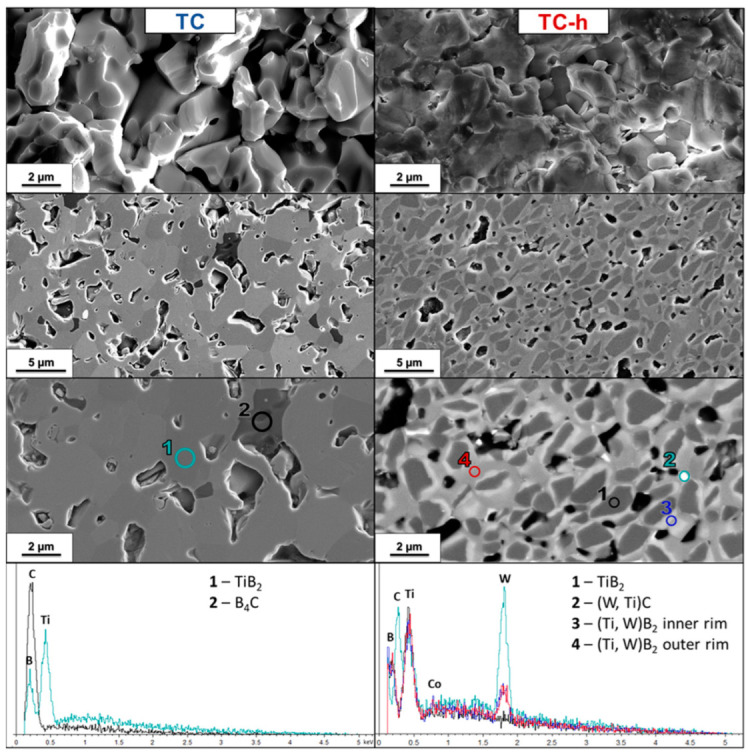
SEM micrograph of TiB_2_ with 5 vol.% B_4_C soft homogenized (TC, **left column**) and high-energy-milled (TC-h, **right column**) samples’ fractured and polished sections and EDS spectrum of polished sections.

TC and TC-h. TiB_2_ without sintering aids (T) did not sinter; in fact, as can be seen in Figure 6, no necks formed between the grains. Adding 5 vol.% B_4_C (Figure 7) improved sintering and densification rate, but after 15 min at 1900 °C, although the necks had already formed, the pores were still interconnected; therefore, the material was in an intermediate stage where pores were closing, and the relative density was less than 80%. With the additional aid of high-energy milling, a microstructure with closed porosities and a relative density of around 89% was reached.

Although XRD spectra did not record the presence of residual crystalline B_4_C, from the polished surface SEM images of TC sample it is possible to notice a dark phase which the EDS spectrum confirmed to be boron carbide (Figure 7). In the HEM sample (TC-h) B_4_C is not present, and wider rims than other HEM samples can be noted. Moreover, EDS revealed that the white contrasting phases in TC-h micrographs are WC with a lower Co content and not WB.

The core rim microstructure had two types of rims: an inner one of a lighter color, therefore heavier atomically, and an outer one of a darker gray color. The EDS spectra of these rims showed no significant differences in the composition.

**Figure 8:**
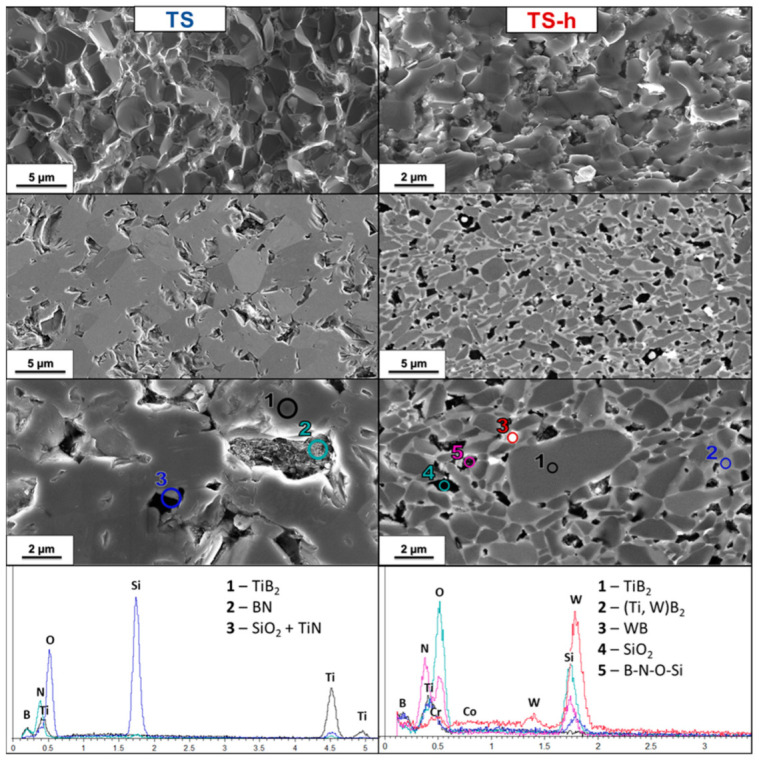
SEM micrograph of TiB_2_ with 5 vol.% Si_3_N_4_ soft homogenized (TS, **left column**) and high-energy-milled (TS-h, **right column**) samples’ fractured and polished sections and EDS spectrum of polished sections.

TS and TS-h. The SEM micrograph of the TS (5 vol.% Si_3_N_4_) polished surface shows a bulk with 14% residual porosity; however, the SEM image of the fracture appears to be totally dense and without any porosity (Figure 8). This is due to the large presence of amorphous compounds like BN and SiO_2_ products from the reaction between Si_3_N_4_ and oxide phases (B_2_O_3_ and TiO_2_).

Porosities in the TS sample’s polished section reveal the presence of secondary phases when observed with the InLens mode. In detail, phases of BN were recognized, with the characteristic lamellar structure, SiO_2_, a darker phase, and TiN, which has a higher molecular weight than TiB_2_, appearing as a light gray phase in the SE2 mode. These phases were confirmed by EDS spectra (Figure 8). The SEM micrograph of the HEM sample with 5 vol.% Si_3_N_4_ (TS-h) showed the typical core–shell structure of TiB_2_ with WB inclusions in addition to the phases already seen in the soft homogenized sample. Despite the presence of WC contamination in the powders which reacted with the oxide phases to form WB, the formation of SiO_2_ and BN phases can still be noted. In addition, an amorphous phase containing B, O, N and Si was identified among the grains.

TM and TM-h: As already observed for TS, the TM sample (with 5 vol.% MoSi_2_ soft homogenized) also does not have empty pores, but silica pockets produced by the reaction between MoSi_2_ and B_2_O_3_, a solid solution containing Mo instead of W with TiB_2_, were formed, leading to a similar core–shell structure to that of the HEM sample. No MoB was observed, but XRD and EDS confirmed the presence of unreacted MoSi_2_, which appears as a white phase.

**Figure 9:**
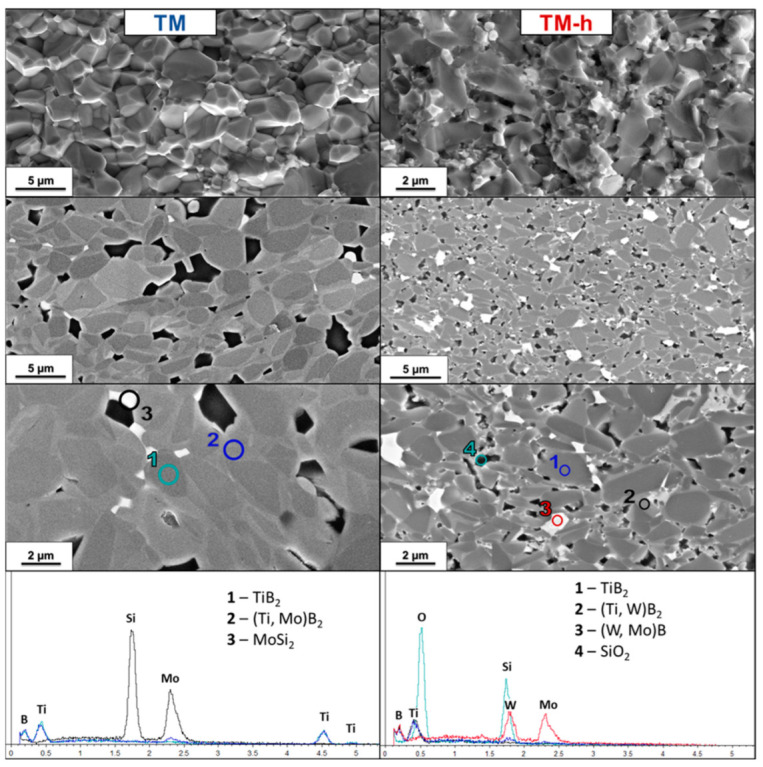
SEM micrograph of TiB_2_ with 5 vol.% MoSi2 soft homogenized (TC, **left column**) and high-energy-milled (TC-h, **right column**) samples’ fractured and polished sections and EDS spectrum of polished sections.

In the HEM sample (TM-h), WC and MoSi_2_ phases combine, resulting in a core–shell structure where the cores of TiB_2_ are surrounded by a rim composed of a solid solution of mixed boride of Ti, W, Mo (and Co in the minor part) with inclusions of mixed borides of W and Mo (and Co). Again, complete densification of the matrix is interrupted by the formation of silica pockets (Figure 9). The rims in this sample are very narrow, and light gray colored TiSi phases can be observed.

#### Densification Mechanisms

The densification behavior was highly affected by the sintering aid and the high-energy milling procedure. Starting from the as-received powder, the effect of milling with WC media was twofold: on one hand, high-energy milling reduced the mean particle size, activating the particle surface; on the other, contamination from WC led to elimination of surface oxides through surface reactions on the TiB_2_ particles (TiO_2_ and B_2_O_3_) [46,60,61].

The most probable reactions are [61,62,63]:

TiO_2 (s)_ + 3WC _(s)_ → TiC _(s)_ + 3W _(s)_ + 2CO _(g)_ (1)

3TiB_2 (s)_ + TiO_2 (s)_ + 6WC _(s)_ → 4TiC _(s)_ + 6WB _(s)_ + 6CO _(g)_ (2)

3WC _(s)_ + B_2_O_3 (s)_ → 2WB _(s)_ + W + 3CO _(g)_ (3)

WC particles favored the elimination of surface oxides, while release of W resulted in the formation of core–shell structures in TiB_2_. Although the formation of TiC seems favored, it was not detected with EDS and XRD methods. The large presence of WB inclusions and the absence of TiC therefore suggest a greater relevance of Reaction (3) between WC and B_2_O_3_.

The addition of sintering aids made the picture even more complex with other reactions taking place. In addition to Reactions (1)–(3), B_4_C reduced the oxygen-carrying chemical species present on the surfaces of the TiB_2_ powder particles through the following reaction:

7TiO_2 (s)_ + 5B_4_C _(s)_ → 7TiB_2 (s)_ + 5CO _(g)_ + 3B_2_O_3 (l)_ (4)

WC inclusions can also react with B_4_C via the reaction:

2WC _(s)_ + B_4_C _(s)_ → 2WB_2 (s)_ + 3C _(s)_ (5)

The main effect of Si_3_N_4_ was to remove surface oxides from TiB_2_ powder particles and form liquid phases according to [64]:

Si_3_N_4 (s)_ + 2B_2_O_3 (l)_ → 4BN _(s)_ + 3SiO_2 (l)_ (6)

2Si_3_N_4 (s)_ + 6TiO_2 (s)_ → 6TiN _(s)_ + 6SiO_2 (l)_ + N_2 (g)_ (7)

2TiB_2 (s)_ + 3N_2 (g)_ → 2TiN _(s)_ + 4BN _(s)_ (8)

Subsequently, the liquid formed at about 1600 °C was very likely to be due to the reaction of Si_3_N_4_ remaining from Reactions (6) and (7) with SiO_2_ and TiB_2_. During cooling, BN, TiN and SiO_2_ pockets precipitated from the liquid phase (Figure 8), but some of them were retained along the grain boundaries in form of amorphous Si-O-B-N-Ti films.

Similar to other borides, for MoSi_2_ addition, densification is presumed to be assisted by a transient liquid phase, due to the reaction between MoSi_2_ and surface oxide impurities on the diboride particles [65,66]. Several reaction paths were indicated, as suggested by the variety of crystalline and amorphous phases identified in the dense microstructures. The presence of crystalline MoB (only for the HEM sample) and pockets of amorphous SiO_2_ suggests the following reaction occurs:

2MoSi_2 (s)_ + B_2_O_3 (l)_ → 2MoB _(s)_ + 2.5Si _(l)_ + 1.5SiO_2 (l)_ (9)

Reaction (9) implies the removal of B_2_O_3_ from boride particle surface and formation of Si- or Si-O-based liquids. According to the Mo-Si-B phase diagram (Figure S1) [67], liquids could form at temperatures as low as 1350 °C. The formation of liquid promotes mass transfer mechanisms via partial dissolution of the boride matrix. The observed formation of epitaxial core–shell structures suggests a solution re-precipitation mechanism even if diffusion of Mo into the diboride lattice could also occur. During cooling, the transient liquid phase solidifies, resulting in the formation of discrete crystalline phases.

The mixing of TiB_2_, WB and MoB resulted in a core–shell microstructure (Figure 9) with TiB_2_ core, a mixed boride rim with the three metals (Ti, W, Mo)B_X_ and (W, Mo)B_X_ particles.

### 3.3. Mechanical Properties

Hardness and fracture toughness measurements were conducted for all the samples except T and TC, as their density was below 80% (refer to Table 1). The hardness of the samples was negatively influenced by porosity and the presence of softer phases, while it increased with a decrease in the mean grain size. In this batch of materials, the highest hardness value, 24.5 GPa, was observed for T-h, indicating that high-energy milling (HEM) has a comparable effect on hardness as the addition of additives. Moreover, both samples with the addition of MoSi_2_ (TM and TM-h) exhibited similar hardness values due to their higher relative density and relatively small mean grain size. The lowest hardness value, 20 GPa, was recorded for TC-h, which had the highest porosity among the high-energy-milled samples.

Also, fracture toughness increased with the decrease in grain size and increase in density. However, it was sensitive to the presence of defects, secondary phases and their dispersion in the material. The highest fracture toughness value, 5.4 MPa m^1/2^, was measured for TS and TM samples, which were soft homogenized with Si_3_N_4_ and MoSi_2_, respectively. These samples exhibited large pockets of silica embedded within the material. T-h also showed a similar level of toughness, while the HEM samples with aids displayed lower values, approximately 4 MPa m^1/2^ or below, despite their high density.

The highest hardness and fracture toughness values obtained in this study are comparable to the limits reported in the literature for TiB_2_ samples with very high density, see Table 2, achieved through various sintering techniques such as hot pressing, spark plasma sintering and microwave sintering.

### 3.4. Optical Properties

The optical properties were measured for selected samples, e.g., T, T-h, TS and TC.

**Figure 10:**
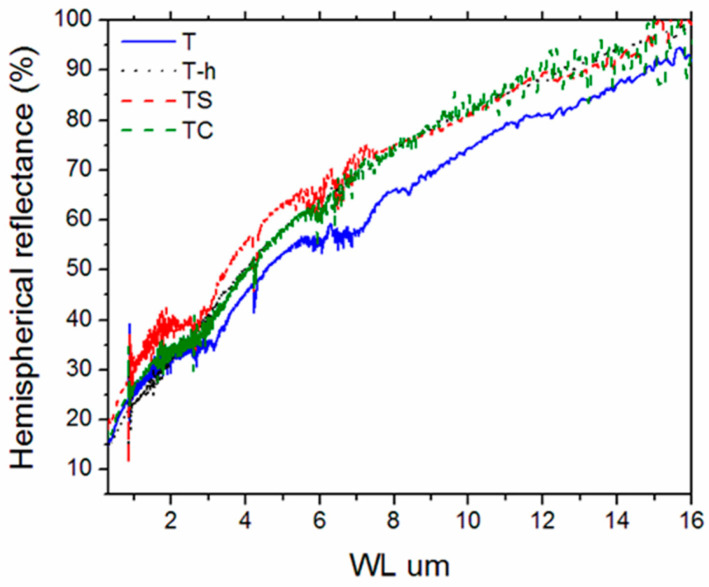
Experimental spectral hemispherical reflectance of the sintered sample of TiB_2_ as received (blue line), high-energy-milled (black line) and with 5 vol.% of Si_3_N_4_ (red line) or B_4_C (green line).

From optical spectra (Figure 10), it can be seen that all samples keep the original trend of the pure and dense TiB_2_ matrix [53], with the increase in reflectance values from UV–visible to the mid-infrared (MIR), although the step-like curve of the latter appears to have translated into more smoothly increasing curves. The reason can be primarily ascribed to the lower density of the samples investigated in the present work. In fact, the distorting effect of porosity on the reflectance curves of materials has been reported in the literature [70,71,72]. This is also confirmed if T, T-h and TS spectra are compared.

The densest (T-h) sample shows the smoothest curve, while the sample T is the most porous and complex one within this group, and the TS sample shows intermediate porosity and behavior. On the other hand, the presence of (W, Co, Cr)B_X_ phases and a (Ti,W)B_X_ solid solution detected by EDS in the T-h sample does not seem to significantly affect the T-h spectrum, as qualitatively similar spectral features (the shoulder around 2.6–3 μm wavelength) are also detected in the spectrum of TS, which was not subjected to high-energy milling.

From the experimental hemispherical reflectance spectra, it is possible to estimate the solar absorptance α and the thermal emittance ε(T) at the temperature T, as widely accepted in the literature [73], according to the following relationships:

(10) $$\alpha =\frac{\int_{\lambda_{min}}^{\lambda_{max}}(1-{\rho^{\cap }}^{}\left(\lambda \right))\cdot S\left(\lambda \right)d\lambda }{\int_{\lambda_{min}}^{\lambda_{max}}S\left(\lambda \right)d\lambda }$$

(11) $$\varepsilon =\frac{\int_{\lambda 1}^{\lambda 2}(1-{\rho^{\cap }}^{}\left(\lambda \right))\cdot B\left(\lambda ,T\right)d\lambda }{\int_{\lambda 1}^{\lambda 2}B\left(\lambda ,T\right)d\lambda }$$

where ρ^∩^(λ) is the experimental spectral hemispherical reflectance, S(λ) the AM 1.5 solar spectrum [74], B(λ,T) is the blackbody spectral radiance at the temperature T of interest and the integration bounds are λ_min_ = 0.3 µm and λ_max_ = 3.0 µm for Equation (10) and λ1 = 0.3 µm and λ2 = 16.0 µm for Equation (11). It is worth noting that, being obtained form room-temperature spectra, the values obtained from Equations (10) and (11) can be seen as estimated values, useful for a comparative evaluation of samples when they do not undergo chemical or physical changes at the temperatures of interest [75]. The comparison is particularly robust if the samples are quite similar to each other, e.g., they belong to the same family or they have compositions with minor differences, like in our case. As for the discrepancy we could expect between the calculated and experimental values of at high temperatures, from the literature it is possible to infer that ε is generally underestimated (see for instance [55,76]).

**Figure 11:**
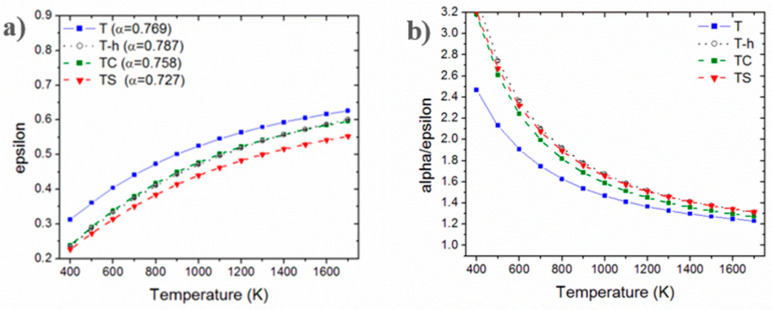
Calculated hemispherical emittance (**a**) and spectral selectivity (**b**) of the sintered sample of TiB_2_ as received (blue line/symbols), high-energy-milled (black line/symbols) and with 5 vol.% of Si_3_N_4_ (red line/symbols) or B_4_C (green line/symbols).

The temperature-dependent emittance curves are shown in Figure 11a; the solar absorptance spectra are indicated in the legend, while the spectral selectivity α/ε is shown in Figure 11b.

If the solar absorptance is considered, the sample showing the highest values is T-h. It has an α value of around 0.79, which is an excellent value compared to that of the reference material in most advanced solar absorbers, i.e., SiC [57]. For all samples investigated here, α is significantly higher than that of the dense TiB_2_ previously reported [53].

For all samples, the estimated thermal emittance increases with temperature due to the interplay between the changes in the spectral distribution of the blackbody radiation as the temperature increases and the shape of the spectral reflectance of samples. In particular, as the temperature increases, the peak wavelength of the blackbody radiation is moved towards shorter wavelengths, where the samples’ reflectance is lower (Figure 11), arising in a higher value of the ratio in Equation (11). The increase in ε(T) with temperature is thus strictly related to the optical properties of the material under investigation. In the majority of cases, the emittance increases with temperature like in this case but not always (see for instance the materials investigated in [77]).

Spectral selectivity α/ε is one of the figures of merit that can be used to evaluate the characteristics of a solar absorber [78]. It gives information about the capability of the material to retain the thermal energy collected through the sunlight absorption before thermal radiation intervenes to decrease the collected energy. As such, a higher α/ε value is desired, providing that, for maximizing the absorber efficiency η_abs_, it is not achieved through an insufficient α. From Figure 11b, the samples with the highest α/ε are T-h and TS, among which T-h can be considered as the most promising one due to its significantly higher solar absorptance. On the other hand, T-h favorably compares to TC as well due to the better α value, despite the similar emittance.

## 4. Conclusions

TiB_2_ ceramics were prepared through hot-pressing sintering after ball milling or high-energy milling using WC-Co milling media for four different compositions: TiB_2_ as received and with 5 vol.% of B_4_C, Si_3_N_4_ or MoSi_2_ as a sintering aid. The addition of sintering aids, except for B_4_C which only had a slight improvement in sintering, accelerated the densification process but also resulted in the formation of secondary phases. Specifically, a liquid phase was formed at around 1600 °C when using Si_3_N_4_ or MoSi_2_ as aids, which improved the densification rate but left silica pockets within the bulk material after sintering.

High-energy milling proved to be highly effective in reducing the size of the starting powders, leading to improved densification. The presence of carbon from WC doping exerted a cleaning action, contributing to elimination of surficial oxide phases. W remnants were incorporated in the TiB_2_ lattice, resulting in the formation of core–shell structures, the development of which was influenced by temperature and holding time.

Even without the addition of sintering aids, high-energy milling of TiB_2_ alone resulted in a density of 93.4% and the highest recorded Vickers hardness of 24.5. The best material was the one with added 5 vol.% MoSi_2_ and high-energy-milled, which achieved almost complete densification (although limited by the presence of silica pockets) at 1700 °C. This material also exhibited a Vickers hardness of 24.4 GPa, indicating its superior hardness properties.

Optical properties were measured for T, T-h, TS and TC. T-h showed both the highest α value around 0.79, and the highest spectral selectivity α/ε, indicating additive-free high-energy-milled TiB_2_ as the most promising sample.

## Figures and Tables

**Table 1:** Sintering parameters, microstructural features and mechanical properties of hot pressed and pressureless sintered TiB_2_ composites.

Label	Sintering Aid	HEM	Max T/Time	Bulk Density	Relative Density	Crystalline Phases	Mean Grain Size	HV 1.0	K_Ic_
	vol.%	No	°C/min	g/cm^3^	%		TiB_2_, μm	GPa	MPa m^1/2^
**T**	No	No	1950, 20	3.45	≈76	TiB_2_	3.2 ± 0.1	-	-
**TC**	5 B_4_C	No	1900, 15	3.71	≈78	TiB_2_	2.4 ± 0.1	-	-
**TS**	5 Si_3_N_4_	No	1900, 15	4.28	85.6 * (>98)	TiB_2_, TiN	3.1 ± 0.1	22.2 ± 0.5	5.4 ± 0.4
**TM**	5 MoSi_2_	No	1900, 10	4.43	95.1 * (>98)	TiB_2_, MoSi_2_	1.8 ± 0.1	24.4 ± 0.4	5.4 ± 0.2
**T-h**	No	WC media	1900, 10	4.60	93.4	TiB_2_, WB, W_2_CoB_2_	1.0 ± 0.1	24.5 ± 0.2	5.0 ± 0.5
**TC-h**	5 B_4_C	WC media	1900, 10	4.22	87.9	TiB_2_, WB	1.3 ± 0.1	19.6 ± 0.3	4.1 ± 0.1
**TS-h**	5 Si_3_N_4_	WC media	1830, 10	4.54	92.4 * (>98)	TiB_2_, TiN, WB	0.9 ± 0.1	23.2 ± 0.2	3.6 ± 0.1
**TM-h**	5 MoSi_2_	WC media	1700, 10	4.67	94.7 * (>98)	TiB_2_, TiC, TiSi, MoB, WB	0.8 ± 0.1	24.4 ± 0.2	3.5 ± 0.1

* The sample has pockets of silica that appear as porosity (the actual density is reported in brackets).

**Table 2:** Sintering parameters, microstructural characteristics and mechanical properties of TiB_2_ samples sintered with different sintering methods and different amounts of additives. Notes: HP—hot pressing, MWS—microwave sintering, SPS—spark plasma sintering.

Sample Composition	Processing Details: Method, Temperature, Dwell Time	Relative Density	Mean Grain Size	HV 1.0	K_Ic_	Reference
wt%	°C/min	%	TiB_2_, μm	GPa	MPa m^1/2^	
TiB_2_	HP, 1800, 60	90	-	24	5.8	Park et al. [64]
TiB_2_	MWS, 1700, 10	95.5	2.0	24	5.6	Demirskyi et al. [68]
TiB_2_	SPS, 2000, 5	96	6–10	23.5	5.5	Demirskyi and Sakka [69]
TiB_2_	HEM, HP, 1900, 10	93.4	1.0	24.5	5.0	Present work
TiB_2_ + 2.5Si_3_N_4_	HP, 1800, 60	99	-	27	5.1	Park et al. [64]
TiB_2_ + 10Si_3_N_4_	HP, 1800, 60	96	-	20	5.4	Park et al. [64]
TiB_2_ + 3.6Si_3_N_4_	HP, 1900, 15	>98 *	3.1	22.2	5.4	Present work
TiB_2_ + 3.6Si_3_N_4_	HEM, HP, 1830, 15	>98 *	0.9	23.2	3.6	Present work
TiB_2_ + 6.8MoSi_2_	HP, 1900, 10	>98 *	1.8	24.4	5.4	Present work
TiB_2_ + 6.8MoSi_2_	HEM, HP, 1700, 10	>98 *	0.8	24.4	3.5	Present work
TiB_2_ + 10MoSi_2_	HP, 1700, 60	99.3	1–2	27	4.0	Ch. Murthy et al. [7]
TiB_2_ + 20MoSi_2_	HP, 1700, 60	98.7	1–2	25	5.0	Ch. Murthy et al. [7]
TiB_2_ + 10TaC	SPS, 2000, 5	>98	2–4	28.8	5.9	Demirskyi and Sakka [69]

* The sample has pockets of silica.

## Data Availability

The raw/processed data required to reproduce these findings cannot be shared at this time due to technical or time limitations.

## Supplementary Materials

The following supporting information can be downloaded at: https://www.mdpi.com/article/10.3390/nano13192683/s1, Figure S1: Melting diagram of Mo–Si–B system [67].
