# Supplementary figures and images for: Sintering, Mechanical and Optical Properties of TiB2 Composites with and without High-Energy Milling
Source: Nanomaterials (Basel). 2023 Sep 30;13(19):2683. doi: 10.3390/nano13192683 (PMC10574485; doi:10.3390/nano13192683)

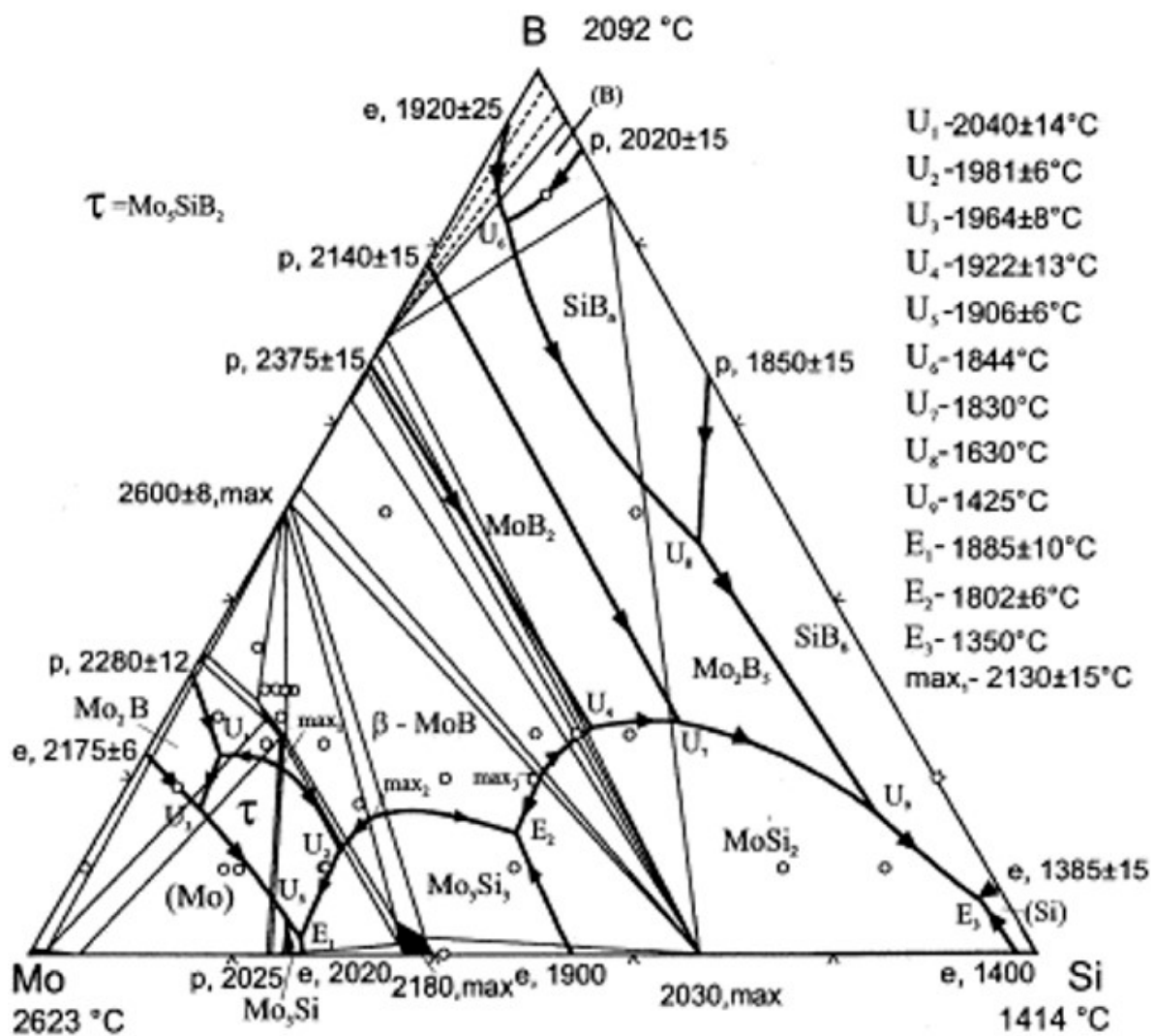

Supplement: Supplementary file 1 [file nanomaterials-13-02683-s001.zip › nanomaterials-2628844-supplementary.pdf]
